# A Conceptual Framework for Modelling Safe Walking and Cycling Routes to High Schools

**DOI:** 10.3390/ijerph17093318

**Published:** 2020-05-10

**Authors:** Mohammad Lutfur Rahman, Antoni Moore, Melody Smith, John Lieswyn, Sandra Mandic

**Affiliations:** 1 Active Living Laboratory, School of Physical Education, Sport and Exercise Sciences, University of Otago, PO Box 56, Dunedin 9054, New Zealand; sandra.mandic@otago.ac.nz; 2 School of Surveying, University of Otago, PO Box 56, Dunedin 9054, New Zealand; tony.moore@otago.ac.nz; 3 School of Nursing, Faculty of Medical and Health Sciences, The University of Auckland, Private Bag 92019, Auckland 1142, New Zealand; melody.smith@auckland.ac.nz; 4 Senior Transport Planner, ViaStrada Ltd., 284 Kilmore Street, Christchurch Central City, Christchurch 8011, New Zealand; john@viastrada.nz; 5 Centre for Sustainability, University of Otago, PO Box 56, Dunedin 9054, New Zealand

**Keywords:** school, safe route, active transport, walking, cycling, built environment, traffic safety, framework, adolescents

## Abstract

Active transport to or from school presents an opportunity for adolescents to engage in daily physical activity. Multiple factors influence whether adolescents actively travel to/from school. Creating safe walking and cycling routes to school is a promising strategy to increase rates of active transport. This article presents a comprehensive conceptual framework for modelling safe walking and cycling routes to high schools. The framework has been developed based on several existing relevant frameworks including (a) ecological models, (b) the “Five Es” (engineering, education, enforcement, encouragement, and evaluation) framework of transport planning, and (c) a travel mode choice framework for school travel. The framework identifies built environment features (land use mix, pedestrian/cycling infrastructure, neighbourhood aesthetics, and accessibility to local facilities) and traffic safety factors (traffic volume and speed, safe road crossings, and quality of path surface) to be considered when modelling safe walking/cycling routes to high schools. Future research should test this framework using real-world data in different geographical settings and with a combination of tools for the assessment of both macro-scale and micro-scale built environment features. To be effective, the modelling and creation of safe routes to high schools should be complemented by other interventions, including education, enforcement, and encouragement in order to minimise safety concerns and promote active transport.

## 1. Introduction

Physical inactivity and sedentary lifestyles among adolescents represent global public health problems [1], with 80% of adolescents (13–18 years old) worldwide not meeting the minimum physical activity guidelines of at least 60 min of moderate-to-vigorous physical activity per day [2,3]. Adolescence is an important stage of life to change physical activity behaviours for both short-term and long-term health benefits [4]. However, the level of physical activity significantly declines during this period [5,6]. In New Zealand, approximately 61% of adolescents do not to engage in sufficient physical activity to gain health benefits [7]. Active transport to and from school (ATS) provides an opportunity to incorporate physical activity into adolescents’ everyday life [8,9]. ATS has physiological and psychosocial benefits for individuals, as well as environmental and social benefits for communities [10,11,12,13,14,15,16]. However, the global rates of ATS are low and coupled with high rates of motorised transport to/from school, especially by private vehicles [6,17]. Motorised transport to/from school limits opportunities for adolescents to accumulate transport-related physical activity [18].

### 1.1. Safe Routes to School

Most studies examining walking and cycling routes to school have been conducted in children, rather than adolescents [19,20,21,22,23]. The absence of traffic lights and pedestrian crossings along the school routes discourage children’s walking and cycling to/from school [19]. Greater distances from home to school also increase the likelihood of children travelling on and crossing arterial roads on their route to school, and hence impose further traffic-related safety issues [20]. Parental concerns related to traffic and personal safety associated with ATS at least in part contribute to increasing rates of parents driving their children to and from school [21,22]. Parents identified the routes to school as unsafe due to the absence of footpaths, presence of high traffic volume, speed, and dangerous crossings, and for personal safety issues (e.g., crime) [24,25].

Safe Routes to School (SRTS) is a programme initiated in Odense, Denmark [26] and subsequently implemented in the United States to encourage children to walk and cycle to and from school [27]. SRTS-related interventions such as improved footpaths and road crossing facilities have increased the rate of walking and cycling among primary school children in the United States [28]. For example, primary school children who have travelled through the SRTS intervention areas were more likely to increase their frequency of walking or cycling to school compared to their counterparts (15% vs. 4% increase) [29]. Improvements of footpaths and traffic control as a part of SRTS interventions contributed to a 33% reduction in pedestrian injury in children aged 5 to 19 years [30]. However, the limitation of these studies includes a lack of objectively measured home-to-school distance data, absence of a control group, lack of control for confounding factors (such as demographic characteristics and socio-economic status) and lack of information on the effectiveness of SRTS interventions for adolescents/high school students. In addition, parental time constraints and perceptions of convenience of different transport modes to school also should be considered for the success of SRTS interventions [28]. Despite the effectiveness of the SRTS interventions to increase the rate of children’s walking and cycling to school in the United States [29,30], the effects of such interventions in adolescents remain unknown.

Knowledge about modelling safe walking and cycling routes to school for adolescents is currently limited and there is comparatively little research and guidance on how to encourage more ATS among adolescents compared to children. Such modelling represents predicting the safe routes based on quantifiable factors associated with built environment features and traffic safety. Modelling SRTS may be useful to inform transport planners or policy makers about which routes would be promoted or prioritized and improved. Previous studies have investigated the characteristics of built environment features along the actual and shortest walking and cycling routes between home and school [19,31]. The presence of favourable built environment features and the perception of traffic safety in school neighbourhoods may influence adolescents’ walking or cycling to school as well as parental decisions to allow their adolescents to walk or cycle to school [32,33]. Therefore, these features along walking and cycling routes to school must be assessed [34] and should be taken into account when modelling safe routes to high schools. Modelling safe school routes should also take into account the unsafe areas along the existing common routes to school in the school neighbourhoods [35], and design of drop-off/pick up points within a reasonable walking or cycling distance from school [36].

### 1.2. Research Aim

**Figure 1:**
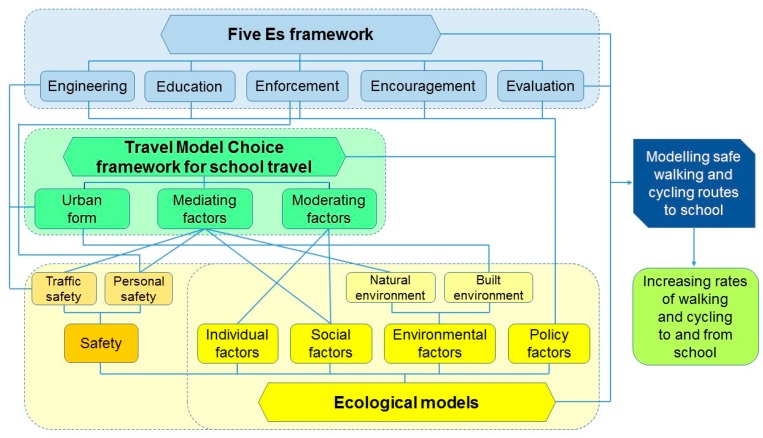
A conceptual framework for modelling safe walking and cycling routes to high schools.

The aim of this study was to develop a new framework for modelling safe walking and cycling routes to high schools (Figure 1). Previously developed frameworks have focused on children and included environmental determinants of active travel framework for children [37], a school travel behaviour framework to assess the association between the child’s ATS and environmental, household, and child factors [38], and travel behaviour framework for children [39]. However, ATS rates and barriers differ in children versus adolescents. Some evidence suggests that ATS rates decline from childhood to adolescence [40,41,42,43,44], which may be in part attributed to increasing distance to high schools compared to primary schools [42]. Another influential factor in declining ATS rates among adolescents is culture (attitudes and behaviour to ATS) [45]. In addition, parental barriers to ATS are influenced by children’s age [46], and may have impact not only on ATS rates but also on levels of independent mobility [47,48]. For example, parents of adolescents in Spain reported more concerns about distance to school and crime and fewer concerns regarding the traffic volume compared to parents of children [46]. Other factors, such as adolescents’ perceptions of safety of walking and cycling to school [32,49,50], their perceptions of the built environment in their home neighbourhood and along the school route [31,51], school choice policies [52], adolescents’ aspirations for motorised transport [53], and reaching the age for obtaining a driving licence may also have effects on how adolescents travel to/from school. Therefore, previously developed frameworks for children may not be transferrable to adolescents.

## 2. Methods

The present study followed a similar methodology for developing a new conceptual framework for modelling safe walking and cycling routes to high schools published elsewhere [54]. The process included a comprehensive review of correlates of ATS among adolescents and the effects safe routes to school interventions on ATS rates among children and adolescents. Literature related to adolescents’ ATS and safe routes to school interventions were searched in ‘’Google Scholar’’, ‘’Scopus’’, ‘’PubMed’’, and ‘’ScienceDirect’’. Some major keywords used for searching and identifying the published articles included ‘‘active transport’’, “school’’, ‘’walking/cycling to school’’, ‘’safe routes to school’’, ‘‘adolescents’’, ‘’interventions’’, and ‘’walking/cycling routes to school’’. Literature related to ATS in adolescents was considered for developing the conceptual framework.

The articles were reviewed to extract significant correlates of adolescents’ ATS in general as well as correlates specific to walking and cycling to school from the literature for developing the conceptual framework. Since evidence related to safe routes to school interventions in adolescents was limited, the relevant literature from primary school children was also considered. Non-peer reviewed journal articles, magazines, newspapers, dissertations, and articles published in languages other than English were not considered. The existing relevant frameworks were also reviewed [37,38,39,55,56,57,58], in order to identify the most important factors to be considered for developing a conceptual framework for modelling safe walking and cycling routes to high school.

The conceptual framework was developed based on the integration of the existing relevant frameworks, including ecological models [55,56,57] with traffic and personal safety considerations, the ‘’Five Es’’ framework of transport planning [58]; and the travel mode choice framework for school travel [39]. Those three frameworks were chosen because together they integrate (a) individual, social, environment, and policy factors that correlate with ATS in adolescents (ecological models) [55,56,57], (b) engineering, education, enforcement, encouragement, and evaluation components used to design safe routes to school interventions to promote ATS among children (Five Es framework) [26,51,58], and (c) urban form and the mediating and moderating factors widely used to explore children’s school travel behaviour (Travel Mode Choice framework) [37,38]. Each of those frameworks is summarised in the subsequent sections.

## 3. Ecological Models for Active Transport to School

Ecological models for ATS account for individual, social, built environment, and policy factors [55,56,57] and take into account traffic and personal safety-related considerations. Correlates of ATS, walking and cycling to school in adolescents are discussed in the subsequent sections and are summarised in Table 1. Positive correlates indicate factors that encourage adolescents more likely to use ATS, whereas negative correlates represent those that discourage adolescents or less likely to use ATS.

### 3.1. Individual Factors

Most previous studies reported that individual factors, such as younger age [59] and male gender [46] are positive correlates of ATS in adolescents. Similar findings were reported in most studies that specifically examined correlates of walking or cycling to school [60,61] (see Table 1 for details). Self-efficacy, another individual factor, was positively correlated with adolescents’ walking and cycling to school [62], but such findings were not observed in other studies that have examined adolescents’ self-efficacy in relation to walking and cycling to school [63,64].

### 3.2. Social Factors

Social factors, such as family support [65], higher parental education [44], and the presence of adult supervision [66] are positive correlates of ATS in adolescents. In contrast, adolescents from higher income families [67] and those living in households with a greater number of vehicles [68] were less likely to use ATS compared to their counterparts. The presence of siblings in a family was not associated with adolescents’ ATS [69]. Positive correlates of both walking and cycling to school among adolescents in most previous studies included social supports, parental education and employment, adult supervision, and a single parent family [46,70]. On the other hand, negative correlates of both walking and cycling to school were living in household of higher income, no siblings in a family, and higher household car ownership [64]. Some studies found no association between the rates of adolescents’ walking or cycling to school and parental employment or number of parents in a family [62,71].

### 3.3. Environment Factors

Environmental factors include both built environment aspects, such as distance, land use mix, street connectivity, intersection density, and neighbourhood aesthetics, as well as natural environment factors, such as topography and climate.

Studies examining built environment correlates of ATS among adolescents reported population density, street connectivity, and neighbourhood aesthetics as positive correlates [32,72]. In contrast, distance to school and intersection density were negative correlates of adolescents’ ATS [36]. Inconsistent results were reported for associations between land use mix in the home neighbourhood and adolescents’ ATS, with some studies reporting a positive association [33,39] and others no association [73]. Home-to-school distance is consistently the strongest predictor of ATS in adolescents [66,68,72]. Threshold distances for adolescents’ walking and cycling to school vary between countries, ranging from 1.4 km to 3.0 km for walking [32,34] and from 3.0 km to 8.0 km for cycling [74,75]. Recent findings also suggest that parental perceptions of adolescents’ walking and cycling to school are changing with increasing distance to school [76]. Findings from the studies that specifically examined correlates of walking and cycling to school showed that population density and street connectivity [62], walking and cycling infrastructure [50], neighbourhood aesthetics [33], and accessibility to local facilities [50] were positive correlates of both modes of ATS, whereas distance to school and higher intersection density were negative correlates [44]. Some studies reported a negative correlation [77] or no correlation [62] between land use mix and adolescent walking and cycling to school.

Natural environment factors, such as cold or unpleasant weather [52,69] and topography (i.e., hills) [40] were negative correlates of adolescents’ walking and cycling to school [46,78]. However, one study found no association between adolescents’ ATS and unpleasant weather [79].

### 3.4. Policy Factors

Policy factors, including school policies, affect walking and cycling to school among adolescents. For example, a lack of school zoning has had negative effects on the rates of ATS among adolescents [52,80,81]. Mandatory use of a bicycle helmet and school uniform requirements were reported as barriers to adolescents’ cycling to school [82,83]. School uniforms represent a barrier for cycling to school particularly among adolescent females [53]. Therefore, relevant school policies should also be considered when planning interventions to increase the rates of walking and cycling to school among adolescents.

### 3.5. Safety Considerations

In addition to individual, social, environmental, and policy correlates of ATS, adolescents’ and their parents’ perceptions of traffic and personal safety of ATS influence decisions about the adolescents’ mode of transport to and from school.

Traffic safety factors, such as traffic volume [32], traffic speed [46], and dangerous intersections [72] were negative correlates of adolescents’ ATS in general as well as negative correlates of walking and cycling to school [84]. In addition, the absence of street lights [40] and safe road crossings along the school routes [50] were negatively associated with adolescents’ walking and cycling to school.

Personal safety factors, such as local crime were negatively associated with adolescents’ ATS [72]. Local crime and the presence of strangers in the neighbourhood were also negatively associated with adolescents’ walking and cycling to school [40,85]. Parental concerns about safety of adolescents’ walking and cycling to school become more pronounced with increasing distance to school [76]. Therefore, traffic and personal safety concerns should be considered and minimised when modelling safe routes for walking and cycling to high schools.

In summary, ecological models suggest that multiple individual, social, environmental, and policy factors, as well as traffic and personal safety concerns of adolescents and their parents, are related to the rates of walking and cycling to school among adolescents. Therefore, ATS-related factors at each level of the ecological models as well as safety considerations must be considered when modelling walking and cycling routes to high schools.

## 4. Five Es Framework

The ‘’Five Es’’ framework of transportation planning [58] includes the following components: engineering (pedestrian and bicycle infrastructure improvement), education (pedestrian and cyclist safety courses), enforcement (increased police patrols or parking enforcement near schools); encouragement (special events or media campaign), and evaluation (data collection and analysis). Modelling and implementation of safe walking and cycling routes to school may use these infrastructure and non-infrastructure related components. For example, the existence of personal safety issues along a route would exclude that route from modelled safe routes for a school. If city neighbourhood or school-based ATS initiatives address personal safety issues along a route, then such routes may be subsequently added into the modelled network of safe walking and cycling routes to a school.

## 5. Framework for Travel Mode Choice to and from School

The framework for travel mode choice to and from school consists of urban form, mediating, and moderating factors [39].

### 5.1. Urban Form

Urban form characteristics include footpaths, crosswalks, and other features of the built environment [39,105]. These characteristics have an impact on decision making around whether youth use a particular route for walking or cycling to school [105]. Most previous studies reporting the results of the interventions related to urban form were conducted in children [19,26,30]. For example, rates of walking and cycling to a primary school increased after children were exposed to interventions, such as the reduction of traffic speed and the relocation of walking from the street or shoulder to the footpaths along the routes to school [26]. The changes in built environment features along school routes, such as the improvement of traffic signals and crosswalk signals, has also encouraged children to walk or cycle to school [30]. The installation of traffic lights and provision of zebra crossings were influential factors for designing a safe route to school, and increased the rate of walking and cycling to school among children in The Netherlands [19]. In adolescents, land use mix, roads with residential and commercial destinations, and cycling lanes separated from traffic correlate with adolescents’ choice of a route for walking and cycling to school [31]. Therefore, urban form factors are associated with walking and cycling to school among children and adolescents, and need to be considered when modelling safe walking and cycling routes to both primary schools and high schools.

### 5.2. Mediating Factors

Mediating factors include factors related to shaping parents’ opinions about the built environment’s support for various transport modes for their child [39,105]. These mediating factors influence the parental decision about whether the route to school is safe for their adolescents to walk or cycle. Therefore, adolescents’ and their parents’ perceptions of the traffic and personal safety along the school routes are affected by crime rates, traffic crash rates, the weather, and the availability of cars and bicycles in a household.

### 5.3. Moderating Factors

Moderating factors include socio-economic status, cultural norms, attitudes, and other factors external to the immediate home environment and the trip to/from school [39,105]. Therefore, moderating factors, such as household socio-economic status, cultural norms, and adolescents’ and their parents’ attitudes towards walking or cycling to school should be considered when modelling safe routes for walking and cycling to high schools.

Taken together, urban form and, mediating and moderating factors are also important for modelling safe walking and cycling routes to high schools. They are related to the decision-making process for travelling to school with adolescents and their parents.

## 6. Proposed Framework for Modelling Safe Routes for Walking and Cycling to School among Adolescents

A proposed conceptual framework for modelling safe walking and cycling routes to high schools integrates the key components of the ecological models for ATS [55,56,57] (including traffic and personal safety perceptions), the Five Es framework of transport planning [58], and the travel mode choice framework for school travel [39] (Figure 1). The proposed framework considers the key components of the existing relevant frameworks and shows how they are interconnected with each other when considered for modelling safe walking and cycling routes to high schools. The framework considers individual, social, environmental (built and natural environments), and policy factors. The framework also integrates both traffic and personal safety factors, acknowledging that some aspects of traffic and personal safety are also linked to social, built environment, and policy factors. Urban form is an important component of travel mode choice framework to and from school and one of the components of the Five Es framework for transportation planning. Built environment features and safety factors are also related to urban form. Individual, social, and natural environment factors (i.e., weather) are inter-connected with moderating and mediating factors of the travel mode choice framework. Policy factors are linked to the engineering, education, enforcement, and encouragement components of the Five Es framework. Policy factors, such as mandatory cycling helmet policy in some countries and rules around e-scooters on roads, may directly impacts adolescents’ school travel modes. The proposed framework also includes the evaluation component of the Five Es framework for transportation planning, which is essential for determining the impact of modelled and constructed safe walking and cycling routes to high schools on the rates of ATS among adolescents.

**Figure 2:**
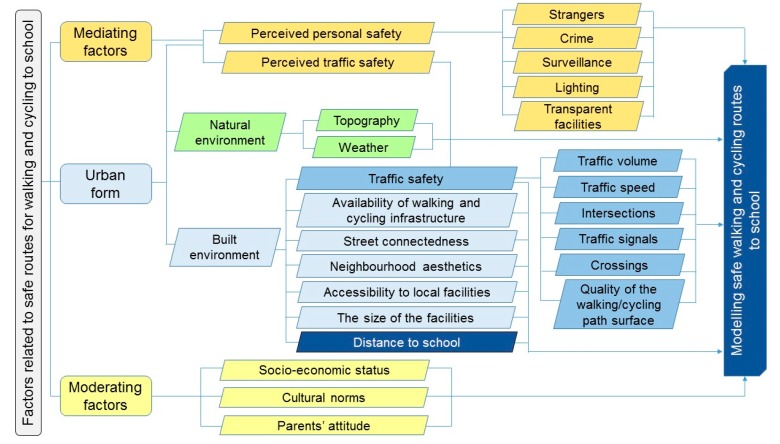
Factors considered for modelling safe walking and cycling routes to school for adolescents.

Specific factors that should be considered when modelling safe walking and cycling routes to high schools are presented in Figure 2. Urban form, as well as mediating and moderating factors derived from travel mode choice framework to and from school [39] should be used as a starting point. Under urban form, the key built environment features that must be considered during the modelling process include reasonable distances for adolescents’ walking and cycling to/from school, infrastructure, street connectedness, neighbourhood aesthetics, accessibility to local facilities (e.g., shops, parks and playgrounds), and the size of the facilities (e.g., big box stores with large parking lots as opposed to small stores). Street connectedness could be measured as the number of intersections per square kilometre, or how well a road network provides direct and short routes to reach destinations [106,107]. Modelling should also consider perceived personal and traffic safety that is related to the urban form such as surveillance, lighting, availability of parking, and the characteristics of local facilities. Surveillance might be ensured by building design (e.g., balconies, front porches, short building setbacks, and back alleys serving as garages), so that people can observe the routes and by streetscape design (e.g., outdoor cafes and the transparency of the facilities, such as clear windows for shops) along the routes to school. Improving passive surveillance is thus important to improve safety and perceptions of safety from crime [106,107,108]. Natural environment factors to be considered include topography and weather. Modelling should also consider traffic safety factors, including traffic volume, traffic speed, number of intersections, presence of traffic signals, safe road crossings, and quality of walking/cycling path surface. Many of the traffic safety factors are related to the built environment features in the home and school neighbourhood, as well as along the school routes. Finally, modelling should also consider ATS-related moderating factors, such as socio-economic status, cultural norms, and adolescents’ and their parents’ attitudes towards walking and cycling to and from school. Taken together, the modelling of safe routes to high schools should address a wide range of built environment features and traffic safety factors, as well as relevant moderating factors within reasonable walking and cycling distances from schools.

## 7. Significance, Implications, and Future Research

The proposed framework for modelling safe walking and cycling routes to high schools extends the current knowledge in that area. Previous studies show that the presence of infrastructure that supports walking and cycling was positively associated with the rates of walking and cycling to school among adolescents [36,46]. Several studies that evaluated SRTS interventions have reported that the implementation of such initiatives increased the rate of children’s walking or cycling to school [27,30]. However, these studies have given more emphasis to education and the encouragement of ATS rather than walking or cycling infrastructure development specifically, and have focused on primary school children rather than adolescents. An assessment of the combined impact of built environment changes, education, and enforcement components could be considered when modelling and evaluating initiatives related to safe walking and cycling routes to high schools.

To inform the modelling of safe walking and cycling routes to school among adolescents, future research should use a combination of multiple tools for the assessment of the built environment, including assessment of both macro-scale features using GIS and micro-scale features (using environmental scans such as the Micro-scale Audit of Pedestrian Streetscapes (MAPS) Global tool [109] or its adaptation for assessing school neighbourhoods [110]). In addition, using Global Navigation and Satellite Systems (GNSS) to collect macro-scale built environmental feature data may provide a valuable additional spatial data source into GIS, alongside shortest path calculations (based on a street network linking geocoded addresses of homes and schools) and digitised drawn routes. For example, two studies reported that adolescents’ cycling routes to school often varied depending on whether the actual or the shortest possible routes were used [31,35]. Adolescents also preferred the shortest routes through residential areas rather than travelling a busy arterial road or crossing it [31,35]. However, the GNSS tracks were not matched with the street network, which may have influenced the study results and underestimated the presence of specific built environmental features along the routes.

In addition, GIS can manage spatial data on the built environment at the macro-scale, used for modelling safe routes to school [111], but such data may not be sufficiently sensitive to identify the micro-scale built environmental characteristics [35,112]. Micro-scale built environmental features along the routes for adolescents’ walking and cycling to school might be assessed using environmental audits, such as the MAPS Global tool [113]. Ideally such audits should be context- specific. For example, an adapted version of the MAPS Global tool [110] was recently used for a detailed investigation of the school neighbourhood built environmental features around high schools in Dunedin, New Zealand [32].

Finally, modelled safe routes should be evaluated prior to implementation, using the context-specific local data. Ideally, the evaluation of modelled routes should also include a prior consultation with the relevant stakeholders, such as local councils and schools, as well as with the potential future users including school staff, adolescents, and their parents, and incorporate their feedback into the final design of modelled routes. To be effective in increasing the rates of adolescents’ walking and cycling to school, modelling and creating safe routes to high schools should also be complemented by other interventions such as education, enforcement, and encouragement in order to minimise perceived traffic and personal safety concerns related to walking and cycling to/from school.

### Strengths and Limitations

A strength of the presented conceptual framework for modelling safe walking and cycling routes to high schools includes an integration of the three relevant existing frameworks related to ATS in adolescents. The presented conceptual framework has also unified these frameworks, resulting in a potentially more widely applicable and valuable framework for modelling safe routes to school. Limitations include using a theoretical approach without testing the proposed framework, and using real-world data and reliance on academic literature from developed countries published in English. Future research should test this framework using actual data in different geographical settings, particularly in settings where walking and cycling to high schools is perceived as unsafe.

## 8. Conclusions

This article introduced a new conceptual framework for modelling safe walking and cycling routes to high schools based on existing literature. The framework suggests that future modelling efforts should focus on addressing the built environment features and factors related to traffic safety. To be effective, modelled safe routes to high schools need to be within reasonable distances for walking and cycling to school in a local context. Ideally, feedback from stakeholders and future users should be sought and incorporated as part of the modelling process. Finalised modelled routes should be also be evaluated prior to implementation, using local data if at all possible. The proposed framework has the potential to assist transport planners and city development authorities to prioritize the tasks and funding related to creating safe walking and cycling routes to high schools. This framework could facilitate future decision-making regarding transport infrastructure investments around high schools including construction and upgrades of walking and cycling infrastructure in the school neighbourhoods to create new safe school routes and improve the safety of the existing walking and cycling routes. With the appropriate planning and design of pick-up and drop-off points within a reasonable walking or cycling distance from school, the existence of safe routes to schools may provide an opportunity for ATS not only for adolescents living within walking or cycling distance to school, but also to those living beyond walking and cycling distances if they use such routes to combine both active and motorised transport modes as part of a single school travel journey.

## Figures and Tables

**Table 1:** Correlates of active transport to and from school in adolescents.

	Active Transport *	Walking to School	Cycling to School
**Individual Factors**			
Age	Positive [44,66,67,68] No correlation [86]	Positive [49,60,61,69,87]	Positive [59,61,63,69] No correlation [62]
Sex (male)	Positive [46,60,67,73] No correlation [18,36,44]	Positive [49] No correlation [69,71]	Positive [49,59,63,84,88,89] No correlation [62,69]
Self-efficacy	-	Positive [34,62]	Positive [34,84] No correlation [63,64]
**Social Factors**			
Peer support	-	Positive [49,88,90]	Positive [63,84]
Family support	Positive [65]	Positive [33,91] No correlation [62]	Positive [33,59,63,84,89,91]
Social support	-	Positive [34,60,62,91]	Positive [34,63,91]
Family Factors			
Household income	Negative [66,67]	Negative [36,61,62,69] No correlation [71]	Positive [61] Negative [44,69] No correlation [62,63]
Parents education	Positive [44,66]	Positive [46,60,69]	Positive [69] No correlation [46,60]
Parents employment	-	Positive [60] No correlation [88]	Positive [60] No-correlation [88]
Adult supervision	Positive [66]	Positive [70]	-
Single parent family	-	Positive [36] No correlation [88]	No correlation [88]
No siblings	No correlation [68]	Negative [92] No correlation [69,88]	Negative [92,93] No correlation [69,88]
Car ownership	Negative [68,73]	Negative [87]	Negative [64]
Bicycle availability	-	-	Positive [18,64]
**Environmental Factors**			
Built Environment			
Distance to school	Negative [46,66,68,72,88]	Negative [34,44,62,87,94,95,96]	Negative [34,39,44,62,74,84,86,88,94,97]
Land use mix (home neighborhood)	Positive [32] Negative [73]	Positive [33,39,50,72,98] Negative [77] No correlation [62]	Positive [33,39,50] No correlation [62]
Population density (home neighborhood)	Positive [32,36,72]	Positive [62,74,77,98,99]	Positive [62,74,99]
Intersection density (home neighborhood)	Negative [32,36]	Negative [97,99]	Negative [97,99]
Direct route to school	-	Positive [88,95,97,98]	Positive [88,95,97]
Walking infrastructure	Positive [36,46]	Positive [32,33,34,48,50,90,91,95,100] No correlation [62]	-
Cycling infrastructure	Positive [46] No correlation [72]	-	Positive [31,32,33,34,36,50,84,91] No correlation [62]
Street connectivity	Positive [33,72,101]	-	Positive [50,62]
Neighborhood aesthetics	Positive [36] No correlation [72,84]	Positive [33] No correlation [62]	Positive [33] No correlation [72,84]
Accessibility to local facilities	-	Positive [33,48,50,65,90,98]	Positive [33,36,50]
School neighborhood walkability index	Positive [32]	-	-
Natural Environment			
Cold weather	Negative [40]	Negative [91]	Negative [91]
Hot weather	Negative [40]	-	-
Unpleasant weather	Negative [46,78]	Negative [69] No correlation [79,102]	Negative [69,102] No correlation [79]
Topography	-	Negative [40,88,103]	Negative [40,88]
**Policy Factors**			
Mandatory use of helmet	-	-	Negative [82,83]
Mandatory wearing of school uniform	-	-	Negative [102,104]
Lack of school zoning policies	-	-	Negative [52]
**Safety Factors**			
Traffic Safety Factors			
Heavy traffic/Traffic volume	Negative [32,46]	Negative [49,88,95,96]	Negative [84,88]
Traffic speed	Negative [46]	Negative [39,50,96]	Negative [39,50] No correlation [84]
No lights in the street	-	Negative [40,89]	Negative [40,88]
Safe road crossing	-	Positive [26,40,50,78,91,96,97]	Positive [40,50,95]
Dangerous intersection	Negative [32,46]	-	-
Evenness of cycling lanes	-	-	Positive [84]
Personal Safety Factors			
Strangers	-	Negative [40,48,85,88,100]	Negative [40,48,85,88]
Local crime	Negative [34,72]	Negative [46,50]	Negative [46,50]

* Studies that have examined active transport to school in general without providing data specific for walking or cycling to school.
